# *PAX* gene expression in autosomal dominant polycystic kidney disease contributes to cyst expansion and regulates a gene network associated with cyst growth

**DOI:** 10.1093/hmg/ddaf205

**Published:** 2026-01-08

**Authors:** Qi Zheng, Cherie Stayner, Glen Reid, Gregory Gimenez, Michael R Eccles

**Affiliations:** Department of Pathology and Molecular Medicine, Dunedin School of Medicine, 56 Hanover Street, University of Otago, Dunedin, 9016, New Zealand; Department of Pathology and Molecular Medicine, Dunedin School of Medicine, 56 Hanover Street, University of Otago, Dunedin, 9016, New Zealand; Department of Pathology and Molecular Medicine, Dunedin School of Medicine, 56 Hanover Street, University of Otago, Dunedin, 9016, New Zealand; Department of Pathology and Molecular Medicine, Dunedin School of Medicine, 56 Hanover Street, University of Otago, Dunedin, 9016, New Zealand; Department of Pathology and Molecular Medicine, Dunedin School of Medicine, 56 Hanover Street, University of Otago, Dunedin, 9016, New Zealand; Maurice Wilkins Centre for Molecular Biodiscovery, Level 2, 3A Symonds Street, Auckland, 1010, New Zealand

**Keywords:** 3D cell culture, autosomal dominant polycystic kidney disease, PAX2, PAX8, RNA-seq

## Abstract

Autosomal dominant polycystic kidney disease (ADPKD) is a common inherited disorder caused by mutations in *PKD1* or *PKD2*. The *PAX2* and *PAX8* genes encode transcription factors required for kidney development, but their role in ADPKD remains unclear. We hypothesized that *PAX2* and *PAX8* contribute to ADPKD cystogenesis through distinct and overlapping mechanisms. Using immunofluorescence, PAX2 and PAX8 expression was assessed in human ADPKD kidney tissues. Nuclear PAX2 expression, and cytoplasmic PAX8 expression were found to be up-regulated in cyst-lining epithelial cells. Furthermore, using siRNA-mediated knockdown of *PAX2* and *PAX8*, significantly reduced 3D spheroid growth was observed in MDCK, WT9–7, and WT9–12 renal epithelial cell line models. RNA sequencing after individual and combined knockdown of *PAX2* and *PAX8* in WT9–7 and WT9–12 cells identified differentially expressed genes and enriched pathways, revealing that *PAX2* regulates pathways related to embryonic development, endoplasmic reticulum stress, and cilia. In contrast, *PAX8* regulates cell cycle and adhesion pathways, while dual knockdown of *PAX2* and *PAX8* impacted extracellular matrix organization pathways. Several genes, including *AGO2* and *WWTR1*, which were previously found to exhibit expression changes in human ADPKD tissues were co-regulated by both factors, suggesting their upregulation in ADPKD may be due to PAX co-expression. Overall, we found PAX2 and PAX8 regulated both unique and overlapping gene networks known to promote ADPKD cystic epithelial growth. Their differential expression patterns and combined impact on extracellular matrix remodelling highlight their potential as therapeutic targets. These findings advance our understanding of ADPKD pathogenesis, and suggest potential avenues for targeted intervention.

## Graphical Abstract

**Graphical Abstract:**
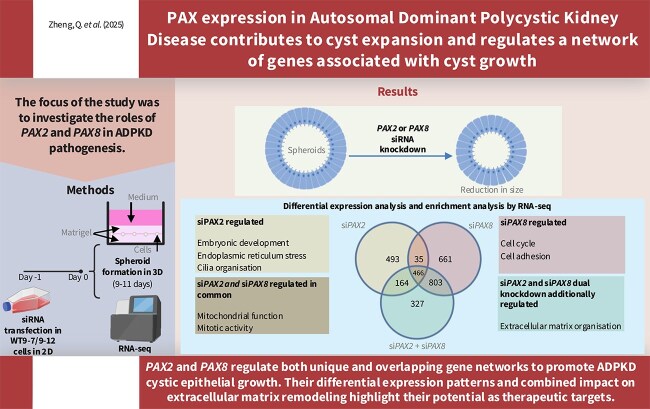

## Introduction

Autosomal dominant polycystic kidney disease (ADPKD) is one of the most common inherited kidney disorders. It is primarily caused by pathogenic variants in *PKD1* or *PKD2*, leading to progressive renal cyst formation, kidney enlargement, and eventual loss of kidney function [1]. Tolvaptan, a vasopressin V2 receptor antagonist, is currently the only approved disease-modifying therapy, but its use is limited by adverse effects such as polyuria and hepatotoxicity [2]. These limitations highlight the need for alternative therapeutic strategies for ADPKD.

The paired box (*PAX*) gene family encodes nine transcription factors essential for embryonic tissue development [3]. Among these, *PAX2* and *PAX8* are key regulators of kidney development. Both share a conserved DNA-binding domain and a C-terminal region that interacts with cofactors, often accompanied by changes in chromatin structure, such as histone methylation, leading to either transcriptional activation or repression depending on the cellular context and cofactor availability [4, 5].

During kidney development, *PAX2* is expressed early in the cap mesenchyme and ureteric bud, maintaining nephron progenitor cells, and promoting mesenchymal-to-epithelial transition (MET) [6]. *PAX8* is expressed slightly later and persists in epithelial segments throughout nephron maturation [6]. In adult kidneys, *PAX2* expression is restricted to parietal epithelial cells and collecting ducts [7], whereas *PAX8* expression remains broadly expressed in tubular epithelium [8]. Both genes have been found abnormally expressed in renal diseases, including renal cell carcinoma and acute kidney injury [3, 9]. Importantly, *PAX2* is re-expressed in ADPKD cyst-lining epithelium, and reducing *Pax2* gene dosage in animal models significantly slows cyst growth, suggesting that *PAX2* reactivation contributes to cystogenesis [7, 10].

Given their overlapping roles in kidney development and potential functional redundancy [11, 12], it is important to investigate the individual contributions of *PAX2* and *PAX8* in ADPKD. In this study, the expression patterns of PAX2 and PAX8 were first assessed in human ADPKD kidney tissues using immunofluorescence staining. Then, siRNA-mediated knockdown of *PAX2* and *PAX8* was performed to investigate their effects on three *in vitro* cell growth models. Finally, RNA sequencing was carried out following individual and combined knockdown of *PAX2* and *PAX8* to identify distinct and overlapping gene expression changes and associated biological pathways involved in ADPKD pathogenesis.

## Results

### Expression of PAX2 and PAX8 in ADPKD kidneys

**Figure 1:**
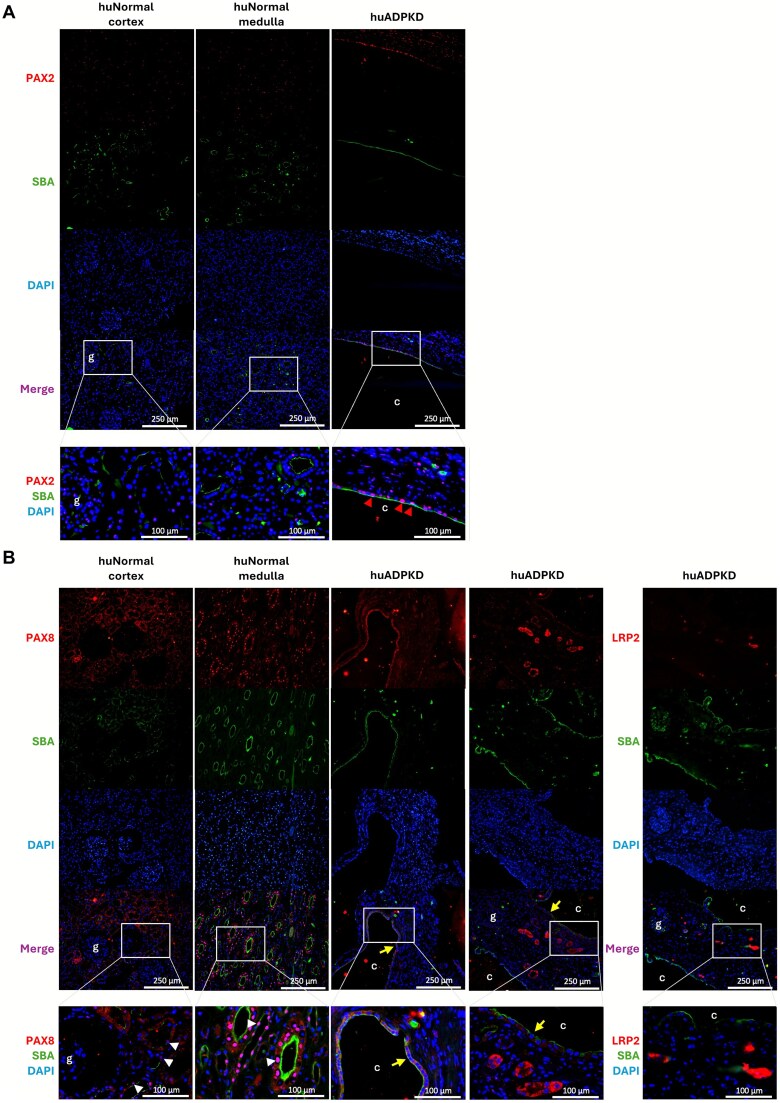
PAX2 and PAX8 expression in human ADPKD. Immunofluorescence staining of PAX2 (A) and PAX8 (B) in human normal renal cortex and medulla, and ADPKD tissues. Representative images showing staining of PAX2, PAX8 or LRP2 (red), with DAPI (blue) for nuclear staining, and SBA lectin (green) labelling renal tubular epithelial cells mainly originated from distal regions. Scale bar = 100 μm or 250 μm. G: Glomerulus. c: Cyst lumen. Red arrowheads in (A) indicate PAX2-positive nuclear staining. Yellow arrows in (B) point cytoplastic staining of PAX8. White arrowheads in (B) point nuclear staining of PAX8.

To investigate PAX2 and PAX8 expression in ADPKD, particularly in cyst-lining epithelial cells, we performed immunofluorescence staining on human ADPKD and normal kidney tissues (Fig. 1). SBA (soybean agglutinins), a marker for distal tubules and collecting ducts [13], strongly labeled cyst-lining cells, while LRP2 (LDL Receptor Related Protein 2), a marker for proximal tubules, was absent, indicating their distal tubule/collecting duct origin (Fig. 1B). In normal adult kidney, PAX2 showed scattered weak nuclear staining, while in ADPKD, nuclear PAX2 signal was increased in SBA-positive cyst-lining epithelial cells (Fig. 1A). PAX8 exhibited weak to strong nuclear staining in proximal and distal tubules and collecting ducts of normal kidneys (Fig. 1B), consistent with previous reports [14]. In ADPKD, cyst-lining epithelial cells exhibited weak cytoplasmic PAX8 staining, rather than the expected nuclear localization, while a similar cytoplasmic pattern was occasionally observed in proximal tubules (Fig. 1B and Table 1).

**Table 1:** Intensity of PAX8 staining in human normal and ADPKD cystic renal tissues.

	Normal		ADPKD	
	Proximal	Distal and collecting ducts	Proximal	Distal and collecting ducts
**Nuclear**	+	+++	−	−
**Cytoplasm**	++	+	+++	+

Note: Proximal tubules were identified by LRP2-positive staining in the cortex (normal tissue: refer to Fig. 1A and B; ADPKD: identified via serial sections). Distal tubules and collecting ducts were identified by SBA-positive staining in the medulla (normal tissue) or SBA-positive and LRP2-negative staining in ADPKD (serial sections). Staining intensity was visually assessed, reflecting qualitative observations from representative fields and does not represent a systematic analysis of all cysts. “−” = no staining; “ + ” = weak positive; “++” = moderate positive; “+++” = strong positive.

As supporting evidence, an analysis of a publicly available snRNA-seq dataset [15] of human ADPKD and control kidney tissues (https://humphreyslab.com/SingleCell/) showed cell-type specific expression differences of *PAX2* and *PAX8* (Fig. S1A-F). Additionally, both *PAX2* and *PAX8* mRNA levels were significantly upregulated in CNT_PC cells (connecting tubule and principal cells, Fig. S1G), which contributes to cysts in this dataset.

### *PAX2* and *PAX8* knockdown reduces spheroid growth in ADPKD cell models

To investigate the roles of *PAX2* and *PAX8* in cyst-like growth, we used siRNA-mediated knockdown approaches targeting each gene in MDCK cells, and in human ADPKD WT9–7, and WT9–12 cells. Real-time quantitative polymerase chain reaction (RT-qPCR) confirmed that si*PAX2* reduced *PAX2* mRNA levels without affecting *PAX8* levels in MDCK and WT9–7 cells, but si*PAX2* treatment had no detectable or significant effect in WT9–12 cells (*P* ≥ 0.05; Fig. S2A), probably due to very low endogenous *PAX2* expression levels in WT9–12 (Fig. S2B). Protein levels of PAX2 showed a similar pattern (Fig. S3A and B). On the other hand, si*PAX8* specifically reduced *PAX8* but not *PAX2* mRNA in all three cell lines, and also reduced PAX8 protein levels (Figs. S2D and E and S3C and D).

**Figure 2:**
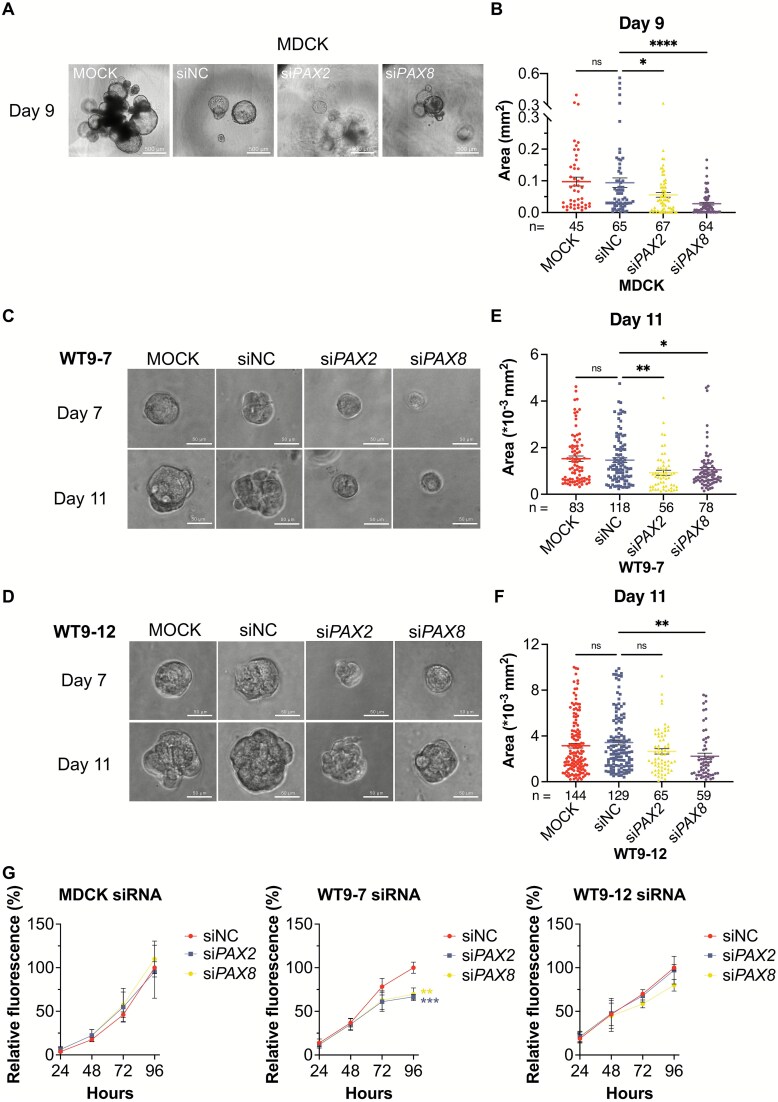
Knockdown of *PAX2* or *PAX8* reduces spheroid growth in ADPKD cells and mildly inhibits cell proliferation in 2D culture. (A) Representative images of MDCK spheroids transfected with siNC, si*PAX2*, or si*PAX8* at day 9 in the presence of 10 μM forskolin. Scale bar = 500 μm. (B) Quantification of spheroid area in transfected MDCK cells at day 9, pooled from ≥3 experiments. (C–D) representative images of WT9–7 and WT9–12 spheroids transfected with siNC, si*PAX2*, or si*PAX8* at day 7 and day 11, respectively. Scale bar = 50 μm. (E–F) quantification of spheroid area in WT9–7 and WT9–12 cells at day 11, pooled from ≥3 experiments. Mean ± SEM. One-way ANOVA with multiple comparisons versus siNC. (G) SYBR green I cell proliferation assay of MDCK, WT9–7, and WT9–12 cells transfected with siNC, si*PAX2*, or si*PAX8* for 4 days in 2D culture. Fluorescence was normalized to siNC at 96 h. mean ± SD from n = 3 biological replicates. One-way ANOVA with multiple comparisons. Ns: Not significant; ^*^P ≤ 0.05; ^**^P ≤ 0.01; ^***^P ≤ 0.001; ^****^P ≤ 0.0001.

Spheroid growth assays showed that both si*PAX2* and si*PAX8* significantly suppressed forskolin-induced MDCK cyst-like spheroid growth (Fig. 2A and B). A similar reduction was observed in WT9–7 spheroids (Fig. 2C and D). In WT9–12 cells, only si*PAX8* resulted in a significant decrease in spheroid size. Although manual visual inspection suggested a slight reduction of si*PAX2*-treated spheroids, the quantification was not statistically significant, consistent with the minimal knockdown of *PAX2* expression (Fig. 2E and F). These results suggest that both *PAX2* and *PAX8* may contribute to cyst-like growth in ADPKD models.

To investigate whether reduced spheroid size was linked to changes in cell proliferation, 2D proliferation assays were performed. Interestingly, WT9–7 cells showed a modest but significant reduction in proliferation after si*PAX2* or si*PAX8* treatment, while WT9–12 cells showed a similar decreasing trend with si*PAX8* (Fig. 2G). This suggests that reduced spheroid growth in WT9–7 and WT9–12 cells may partially be driven by decreased proliferation.

### Distinct and shared pathway enrichment occurs following *PAX2* and *PAX8* knockdowns

Due to the unclear mechanism by which *PAX2* and *PAX8* influence cyst growth, RNA sequencing was performed following siRNA knockdowns in WT9–7 and WT9–12 cells. Due to insufficient *PAX2* RNA levels in WT9–12, knockdown of *PAX2* was undetectable in WT9–12 cells, therefore RNA-seq following siPAX2 knockdown was conducted only in WT9–7 cells. Given the overlapping functions of *PAX2* and *PAX8,* we also included dual knockdown of *PAX2* and *PAX8* (si*PAX*) in WT9–7 cells to assess potential combined effects.

**Figure 3:**
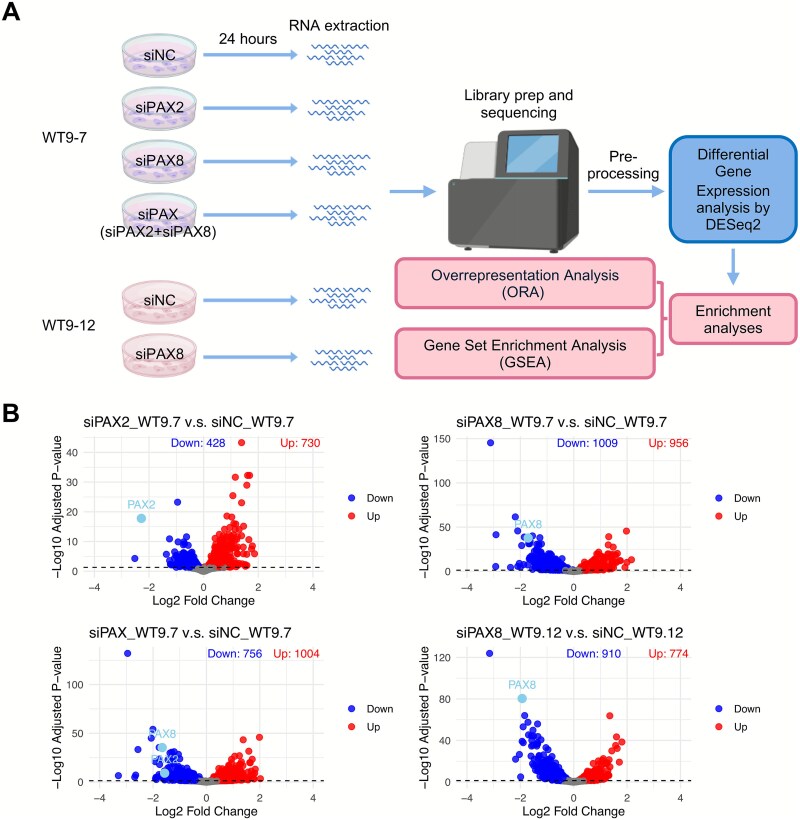
Transcriptomic changes following si*PAX2* and si*PAX8* knockdown in ADPKD cells. (A) Schematic illustration of the RNA-seq experimental design. (B) Differential gene expression analyses for each treatment group (si*PAX2*, si*PAX8*, si*PAX* (si*PAX2* + si*PAX8*) in WT9–7; si*PAX8* in WT9–12) compared to their respective siNC controls. Genes with adjusted P ≤ 0.05 were considered significant. Red: Upregulated; blue: Downregulated; grey: Not significant.

Differential gene expression analysis was performed comparing each knockdown to corresponding siNC control knockdowns, with differentially expressed genes (DEGs) identified using FDR ≤ 0.05 (Fig. 3A and B). Given the modest number of DEGs, with most of them exhibiting ≤ 2-fold change, no additional fold-change cutoff was applied. Nonetheless, the knockdown efficiency was significant, as confirmed by RT-qPCR prior to RNA-seq, and further supported by RNA-seq read counts (Fig. S4).

**Figure 4:**
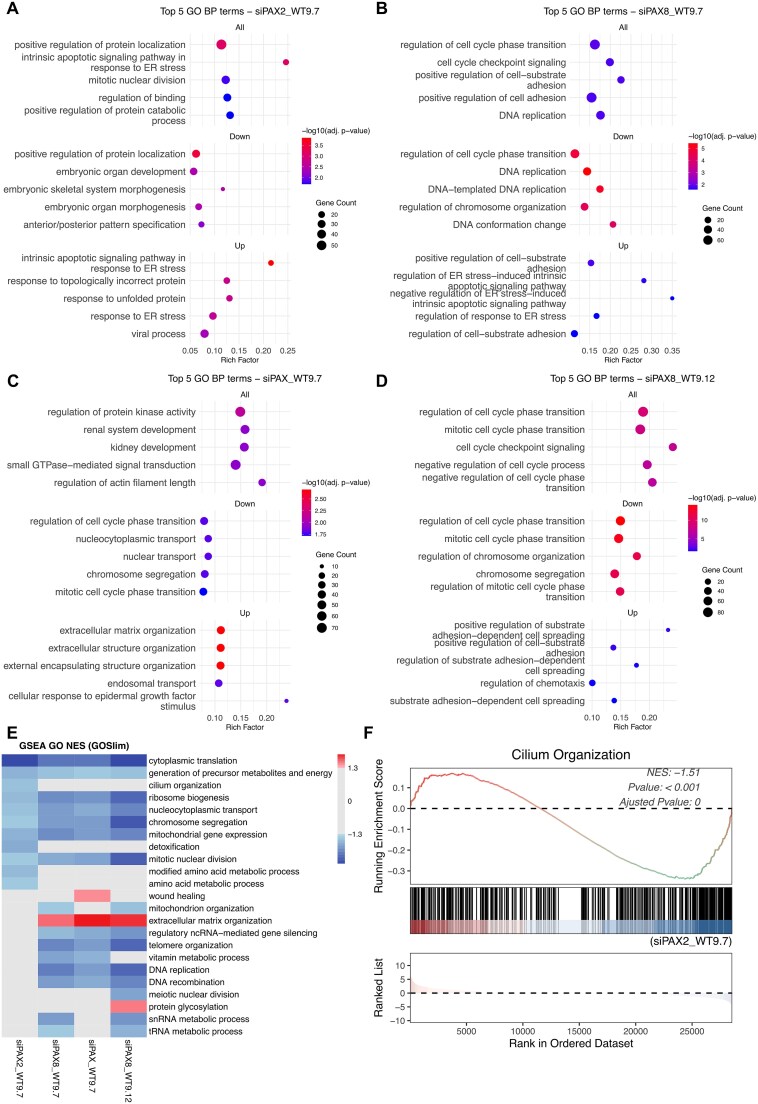
Gene ontology enrichment analysis of *PAX2* and *PAX8* knockdown ADPKD cells. (A–D) over-representation analysis (ORA) of differentially expressed genes in WT9–7 and WT9–12 cells following siRNA-mediated knockdown of *PAX2*, *PAX8*, or both. Enriched gene ontology (GO) biological process (BP) terms are shown for all dysregulated genes (top), as well as separately for upregulated (bottom) and downregulated (middle) genes in each condition: (A) si*PAX2* (WT9–7), (B) si*PAX8* (WT9–7), (C) si*PAX2* + si*PAX8* (WT9–7), and (D) si*PAX8* (WT9–12). Pathways are ranked by adjusted *P* value (FDR ≤ 0.05). (E) Gene set enrichment analysis (GSEA) showing pathways with adjusted *P* ≤ 0.05 in at least one treatment compared to corresponding control (siNC) using GO BP terms filtered by the GO slim subset. Color indicates normalized enrichment scores (NES); grey indicates pathways not significantly enriched (adjusted *P* ≥ 0.05) in that treatment. (F) GSEA result demonstrating negative enrichment of the GO term “cilium organisation” in the si*PAX2* treatment compared to siNC in WT9–7 cells (adjusted *P* = 0.00319).

To investigate altered biological processes, gene ontology (GO) enrichment analyses using both Overrepresention Analysis (ORA) and Gene Set Enrichment Analysis (GSEA) were conducted. ORA using significant DEGs revealed that upregulated genes were enriched in endoplasmic reticulum (ER) stress responses and apoptotic signalling, while downregulated genes were associated with embryonic morphogenesis in WT9–7 cells treated with si*PAX2* (Fig. 4A). *PAX8* knockdown in WT9–7 and WT9–12 consistently influenced pathways related to cell cycle regulation, chromosomal organisation, and cell adhesion (Fig. 4B and D). Overlapping DEGs in both cell lines strongly enriched cell cycle, suggesting a conserved regulatory role for *PAX8* in cell cycle control (Fig. S5). Note, the two ADPKD cell lines were derived from different renal tubule types of the same patient. To further assess whether *PAX2* and *PAX8* co-regulate similar pathways in the same cellular context, we identified genes commonly regulated by both si*PAX2* and si*PAX8* in WT9–7 cells (Fig. S6A) and performed GO enrichment analysis. Limited enrichment was observed only among the downregulated genes, primarily involving developmental and morphogenetic processes (Fig. S6B). Interestingly, these pathways did not overlap with those enriched in si*PAX* treatments.

In si*PAX* (combined *PAX2* and *PAX8*) treated WT9–7 cells, enriched pathways reflected both si*PAX2*-specific (kidney development) and si*PAX8*-specific (cell cycle) effects, while extracellular matrix (ECM) organization was uniquely enriched among upregulated genes, suggesting a potential combined effect of dual knockdown on ECM remodelling (Fig. 4C), which was further supported by enrichment analysis of DEGs uniquely altered with si*PAX* (Fig. S6A and C).

The enrichment analysis of the commonly regulated DEGs between si*PAX2* and si*PAX8* revealed only limited overlap, and individually enriched GO terms of si*PAX2* and si*PAX8* did not intersect using ORA, which focuses only on significantly altered DEGs. Thus, GSEA, which evaluates whether predefined gene sets show systematic differences across the entire ranked gene list, was performed to explore subtle but coordinated changes. To avoid interpretation complexity from thousands of highly specific GO terms, we used GO Slim subset to only capture high-level biological processes. GSEA revealed consistent negative enrichment of pathways related to protein synthesis, mitosis, and mitochondrial function shared across all treatments, which might be commonly regulated by *PAX2* and *PAX8* (Fig. 4E). Additionally, si*PAX2* uniquely affected pathways such as cilium organization (Fig. 4F), while si*PAX8* enriched terms linked to DNA replication and DNA recombination (Fig. 4E). Together, these enrichment analyses suggest that *PAX2* and *PAX8* may have both distinct and overlapping regulatory roles in cystic epithelial biology, while dual knockdown may exert combined effects, particularly in ECM remodelling.

### Distinct and shared gene expression changes occur following *PAX2* and *PAX8* knockdowns

**Figure 5:**
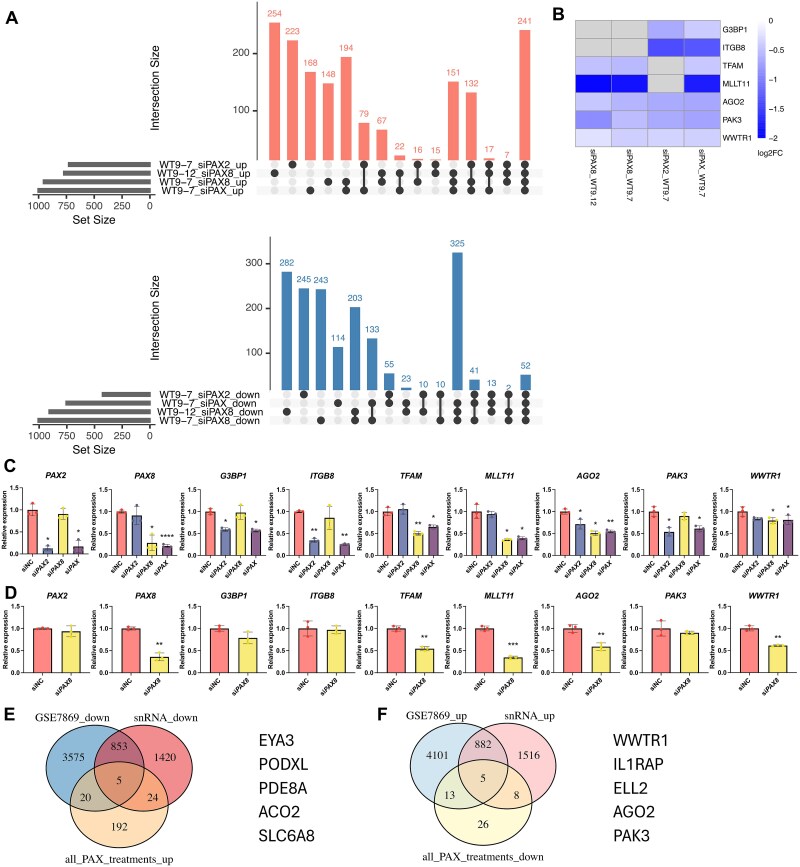
Transcriptomic changes following si*PAX2* and si*PAX8* knockdown in ADPKD cells. (A) UpsetR plots showing overlap of upregulated (red) and downregulated (blue) differentially expressed genes across treatments, shown separately. (B) Heatmap displaying candidate genes for RT-qPCR validation that were specifically downregulated by either si*PAX2* or si*PAX8*, or by both, across WT9–7 and WT9–12 cells. Color represents log2 fold change; grey indicates the gene was not significantly changed (adjusted *P* ≥ 0.05) in that treatment. (C–D) RT-qPCR validation of candidate genes from (B) in WT9–7 (C) and WT9–12 (D) cells. Mean ± SD. One-way ANOVA for (E) and pair t-test for (F). ^*^P ≤ 0.05; ^**^P ≤ 0.01; ^*^P ≤ 0.001; ^****^P ≤ 0.0001. (E-F) integration of RNA-seq results with public ADPKD datasets. Venn diagram (E) showing overlap between genes upregulated following all siRNA treatments and genes downregulated in ADPKD kidney tissue from the GSE3869 microarray dataset and tubular subtypes (PT1, PT2, and CNT_PC) of the published snRNA-seq dataset. *EYA3*, *PODXL*, *PDE8A*, *ACO2*, *SLC6A8* were identified as commonly upregulated following *PAX2* or *PAX8* knockdown but downregulated in ADPKD tissue. Venn diagram (F) showing *WWTR1*, *IL1RAP*, *ELL2*, *AGO2*, *PAK3* were overlapping genes between genes commonly downregulated by all siRNA treatments and genes upregulated in the two ADPKD datasets.

While enrichment analysis revealed pathway-level changes, it does not identify which specific genes are commonly or uniquely regulated by each factor. To address this, UpSet plots were used to distinguish factor-specific and shared effects on gene expression following *PAX2* and/or *PAX8* knockdown (Fig. 5A). To validate RNA-seq findings, RT-qPCR was performed on several downregulated genes, focusing on *G3BP1*, *ITGB8, TFAM*, *MLLT11, AGO2*, *PAK3*, and *WWTR1*, which were chosen because they represent each of the knockdown treatment groups: *G3BP1* and *ITGB8* expression was reduced by si*PAX2* but not si*PAX8*, suggesting *PAX2*-specific regulation; *TFAM* and *MLLT11* were only downregulated by si*PAX8*; *AGO2*, *PAK3*, and *WWTR1* were decreased in all knockdowns and may be co-regulated by *PAX2* and *PAX8*, although there were modest non-significant reductions in PAK3(si*PAX8*) and WWTR1 (si*PAX2*) by RT-qPCR (Fig. 5B-D). Generally, RT-qPCR results were consistent with the RNA-seq data.

Additionally, we sought to identify genes that were altered across all *PAX2*/*PAX8* knockdowns, and which were oppositely regulated in two publicly available ADPKD datasets (a microarray dataset comparing ADPKD cysts to normal kidney tissue, and tubular subtypes (PT1, PT2, CNT_PC clusters) from the snRNA-seq dataset described earlier), reasoning that genes associated with *PAX2*/*PAX8* knockdown and inversely expressed in ADPKD might reflect correlative relationships, potentially highlighting disease-relevant associations. *EYA3*, *PODXL*, *PDE8A*, *ACO2*, and *SLC6A8* were upregulated in all *PAX* knockdown conditions but downregulated in both ADPKD datasets, while *WWTR1*, *IL1RAP*, *ELL2*, *AGO2*, and *PAK3* were downregulated following *PAX* knockdown but upregulated in ADPKD (Fig. 5E and F). These oppositely regulated genes may represent disease-relevant therapeutic targets. However, whether their expression changes occur as a direct consequence of altered *PAX2*/*PAX8* regulation, or whether they occur as a secondary effect, remains to be validated. Notably, several of these genes, such as IL1RAP and WWTR1, are already being explored as therapeutic targets in other diseases, suggesting there might be potential for drug repurposing in ADPKD.

## Discussion

Taken together, our findings suggest that PAX2 and PAX8 may exhibit distinct subcellular localizations in ADPKD cyst-lining epithelial cells and contribute individually to cyst expansion. Transcriptomic analyses indicate that *PAX2* and *PAX8* regulate both unique and shared molecular pathways relevant to ADPKD pathogenesis. These transcription factors and their downstream effectors may represent novel theoretical targets for therapeutic intervention.

Our immunofluorescence results showed nuclear PAX2 in cyst-lining epithelial cells, consistent with previous PAX2 immunohistochemistry investigations [7]. While existing research, together with our results, identified nuclear PAX8 typically in collecting ducts under normal conditions [8, 16], we observed cytoplasmic PAX8 expression with undetectable nuclear PAX8 in SBA-positive, LRP2-negative cyst-lining epithelial cells, indicating that there may be cytoplasmic trapping or novel functions relevant to ADPKD pathogenesis. Given that PAX8 functions as a nuclear transcription factor, the significance of this altered localization remains unknown, although alterations leading to decreased nuclear PAX8 levels, such as for example through reduced nuclear shuttling [17], would theoretically impact PAX8 transcriptional targets through loss of their transcriptional regulation. Similar cytoplasmic PAX8 distribution has been reported in papillary thyroid carcinoma, where it was associated with younger age, larger tumor size, and metastasis [18]. Due to our limited sample size, this observation needs further investigation and validation.

*PAX2* knockdown reduced spheroid growth in all three 3D culture models, consistent with prior *Pkd1*^−/−^mouse studies where *Pax2* heterozygosity limited cyst expansion [7]. This supports the potential of *PAX2* as a therapeutic target. Our proliferation assays suggest that the growth reduction may be partly due to decreased cell proliferation. While the absence of an effect in WT9–12 is inconsistent with the conclusions of the manuscript, these cells are potentially of different tubule-type origin than WT9–7, and *PAX2* is not expressed in all tubule cell types with the potential to form cysts. Our results nevertheless suggest a role for *PAX2* in some tubule types.

*PAX2* and *PAX8* have overlapping roles in several tissues. In acute lymphoblastic leukemia, either gene expression can compensate for *PAX5* loss [11]. Mice lacking *Pax2* exhibit severe renal defects [19, 20], whereas *Pax8*-null mice show normal kidney development [21]. Notably, complete loss of both *Pax2* and *Pax8* results in a failure to form any nephric structures [6], suggesting partial functional compensation during embryonic kidney development. Moreover, in adult kidneys, either single gene knockout does not result in any abnormal phenotype, but combined deletion of *Pax2* and *Pax8* leads to polyuria and impaired urine concentration [12]. However, their relationship has not been investigated in ADPKD, and may have important implications for therapeutic strategies.

This study, including analysis of DEGs and enriched pathways, has revealed potential molecular mechanisms underlying *PAX2* and *PAX8* functions in ADPKD cells. Differentially expressed genes and pathways enriched following *PAX2* knockdown were related to embryonic development, endoplasmic reticulum (ER) stress, and cilia organization. Given the essential role of *PAX2* in kidney development, these pathways may represent reactivation of developmental programs during ADPKD progression. *ITGB8*, downregulated by *PAX2* knockdown, is able to activate latent TGF-β [22], while canonical TGF-β signaling via SMAD2/3 directly suppresses PAX2 transcription [23], suggesting a potential PAX2–ITGB8–TGF-β regulatory loop. Although links between *PAX2* and endoplasmic reticulum (ER) or cilia-related pathways are limited in the literature, their enrichment in this study is interesting given their relevance to ADPKD. ER dysfunction leading to changes in N-linked glycosylation and protein-folding machinery contributes to cyst growth by impairing PC1 trafficking, promoting epithelial apoptosis, and disrupting protein folding [24–27]. Emerging evidence demonstrates that modulating ER chaperone function can restore PC1 stability and ameliorate cyst severity in ADPKD mouse models, indicating that ER stress and its regulation could be a targetable vulnerability in ADPKD, particularly in hypomorphic *PKD1* backgrounds [28]. Although *PAX2* has not previously been implicated in these pathways, our findings raise the possibility that *PAX2* may influence ER homeostasis. *G3BP1*, which was down-regulated following *PAX2* knockdown, is a stress granule nucleator involved in ER stress responses, apoptosis, and the regulation of primary cilia length, and may be a link between *PAX2*, ER stress and cilia organisation [29, 30].

In contrast, *PAX8* knockdown in both WT9–7 and WT9–12 cells enriched pathways associated with the cell cycle and cell adhesion, consistent with roles identified in other contexts, including renal cancer, indicating conserved biological roles [31–33]. Downregulation of *MLLT11*, a cytoskeletal regulator, suggests a role for *PAX8* in supporting cell adhesion [34]. Additionally, *TFAM*, a mitochondrial transcription factor, was downregulated after *PAX8* knockdown. Reduced *TFAM* impairs mitochondrial function, reduces mtDNA content, and increases glycolysis in ADPKD [35]. These results suggest context-dependent roles of *PAX8* in ADPKD.

Several genes with decreased expression in common following either *PAX2* or *PAX8* knockdown, including *AGO2*, *PAK3*, and *WWTR1*, showed elevated expression in ADPKD tissue. Specifically, *AGO2* promotes microRNA-mediated regulation and mitochondrial translation with miR-1 [36]. *PAK3* (which was downregulated in our RNA-seq data, but not significantly in subsequent RT-qPCR) regulates cell cycle progression [37]. *WWTR1*, also known as *TAZ*, drives cyst formation via the TAZ/Wnt/β-catenin/c-MYC axis [38]. Whether these genes are directly or indirectly controlled by *PAX2* or *PAX8* still needs to be confirmed. However, together with enriched processes related to mitochondrial function and mitotic activity observed across knockdowns, and also that some genes we identified, such as *WWTR1*, have been independently identified as being of interest in ADPKD [39], their expression differences and associated pathways may be worth further investigation for developing therapeutic strategies in ADPKD.

Interestingly, dual knockdown of *PAX2* and *PAX8* affected ECM organization, including regulation of multiple collagen subtypes. This aligns with recent findings from AKI models showing that simultaneous deletion of *Pax2* and *Pax8* reduces chronic injury and fibrosis [9]. By day 14 after ischemia–reperfusion injury, collagen I α1 levels and fibrosis markers (e.g. Vcam1 and KIM-1) were significantly lower in *Pax2/Pax8* double knockout mice [9]. This suggests that *PAX2* and *PAX8* cooperatively regulate ECM organisation, and that their dual suppression disrupts a profibrotic transcriptional program. These results offer a theoretical framework that supports the antifibrotic potential of targeting both PAX transcription factors in ADPKD. The mechanism of redundancy or cooperation between *PAX2* and *PAX8* in kidney development and homeostasis remains unclear. The enhanced disruption of ECM-related pathways after simultaneous knockdown of both genes suggests potential cooperative effects. Alternatively, these proteins may regulate overlapping targets through independent mechanisms. A direct molecular interaction between PAX2 and PAX8 hasn’t been reported, and possible cooperative interaction between PAX2 and PAX8 was not directly examined in this study. Nevertheless, several mechanisms could explain a possible cooperative interaction. First, their conserved paired domains allow PAX2 and PAX8 to recognize similar DNA motifs, enabling parallel regulation of shared targets. For example, both factors can bind to and repress *TP53* regulatory elements [40]. Second, their structural similarity outside the paired domain enables recruitment of common co-factors, such as PTIP, which mediates similar H3K4 methylation and chromatin accessibility [41, 42]. It is unknown, however, whether both PAX2 and PAX8 can bind simultaneously to PTIP. Third, PAX2 and PAX8 regulate distinct target genes that converge on common functions: PAX8 controls *SLC14A2*, which affects urea transporters, while PAX2 likely contributes to water and solute transport through other genes such as *AVPR2* [5, 12, 42]. Loss of both impairs urine concentration, showing functional overlap [12].

This study has several limitations. First, the number of immunofluorescence samples used were limited and mutation analysis was not conducted. Quantitative approaches such as whole-slide imaging would be more informative for defining PAX2/PAX8 expression in cysts. Second, limited or no *PAX2* knockdown was observed in WT9–12 cells, likely due to low endogenous expression. Additional models such as alternative ADPKD cell lines, kidney organoids, or *in vivo* mouse models would be suitable as follow-up approaches, especially for investigating ECM regulation. Third, the RNA-seq results revealed relatively modest gene expression changes, and this analysis identified lower than expected numbers of DEGs. This may reflect cell line–specific responses, limited knockdown efficiency, or mild biological effects from partial suppression. Future studies employing CRISPR-based gene knockout models may provide deeper mechanistic insights. Nevertheless, the consistency of our findings with prior literature and the validation of key genes using RT-qPCR support the reliability of our data. Additionally, for gene expression changes validated by RT-qPCR, at least for *TFAM*, publicly available bioinformatic evidence with chromatin immunoprecipitation (ChIP)-seq data (GTRD; https://gtrd.biouml.org/#!) suggests direct binding of *PAX2* or *PAX8* to its promoter regions. Further investigation of direct regulation may involve distal enhancers or indirect mechanisms, which require further experimental validation such as ChIP-seq or ChIP-qPCR.

Given the limited therapies for ADPKD beyond tolvaptan, our findings suggest that modulating *PAX2* and *PAX8*, individually or together, could offer a novel strategy to limit cyst growth and fibrosis. By distinguishing their shared and distinct downstream pathways, our study underscores an important question: whether inhibiting one *PAX* gene, both *PAX* genes, or selectively targeting downstream effectors based on shared or individual regulatory control would be the most effective? The outcome of this question may impact the feasibility of using PAX protein inhibitors, which often tend to act on both PAX2 and PAX8 due to their structural homology [42, 43]. Overall, these results will inform future rational and targeted ADPKD treatment approaches.

## Materials and methods

### Cell culture

Three epithelial cell lines were used: MDCK (Madin-Darby Canine Kidney), WT9–7 (human non-dilated proximal tubule from an ADPKD patient), and WT9–12 (human cystic proximal/distal tubule from an ADPKD patient). WT9–7 cells are heterozygous for a truncating *PKD1* variant (Q2556X), while WT9–12 cells are homozygous for the same variant. All cell lines were obtained from internal laboratory stocks of ATCC-purchased cultures prior to the study.

MDCK cells were cultured in Dulbecco's Modified Eagle Medium (DMEM, Life Technologies, 11995–065) supplemented with 10% fetal bovine serum (FBS; Moregate, BBP5), 1% penicillin–streptomycin (Gibco, 15140–122), and 1% L-glutamine (Gibco, 25030081). WT9–7 and WT9–12 cells were cultured on flasks pre-coated with 0.05 μg/μl Type I collagen and grown in DMEM containing 10% FBS. All cells were maintained at 37°C, 5% CO_2_ with media replaced every 2 days. Cells were passaged at ~ 90% confluence using 0.25% trypsin–EDTA (Gibco, 25200056).

### siRNA transfection

siRNAs (Table S1) were transfected into MDCK, WT9–7, and WT9–12 cells using Lipofectamine RNAiMAX (Invitrogen, 13 778 150) following standard protocols provided by the manufacturer. Final RNA concentrations were 10 nM. Our group has previously shown that *PAX2* knockdown can persist for up to 7 days following si*PAX2* knockdown in bladder and ovarian cancer cells [44]. Transfections were performed in 6-well plates for RNA or protein extraction or 96-well plates for SYBR green I proliferation assay.

### Spheroid formation assay

Matrigel matrix (Corning, 354 234) was thawed overnight on ice at 4°C in the fridge and diluted to 30% (v/v, protein concentration for the Matrigel lot used was 8.9 mg/ml) in FluoroBrite DMEM (Gibco, A1896701) on ice to prepare a working solution. 10 μl of this solution was plated into a 384-well plate (Greiner Bio-One, M1937-32EA), centrifuged at 1000 rpm for one min, and incubated at 37°C for 30 min to form a Matrigel basement layer.

24 h after siRNA transfection, cells were trypsinized and seeded at 20 cells/μl (MDCK) and 50 cells/μl (WT9–7, WT9–12) with 0.22 μm-filtered culture medium. A 2.5 μl droplet of cell suspension was added onto the Matrigel basement, incubated for 20 min, then overlaid with 7 μl of 30% Matrigel. After an additional 30 min, 80 μl of filtered culture medium was added. Medium was refreshed every other day.

Spheroid morphology was imaged at 9 days (MDCK) or 7 and 11 days (WT9–7 and WT9–12) on a BioTek LionHeart FX microscope (Agilent). Spheroid area was quantified using ImageJ.

### RNA extraction and quantitative real-time PCR

Total RNA was extracted from cells 24 h post-transfection using the RNeasy Mini Kit (Qiagen, 74 106) according to the manufacturer’s instructions. RNA concentration was assessed with a NanoPhotometer (IMPLEN, N60).

cDNA synthesis was performed using the High-Capacity cDNA Reverse Transcription Kit with RNase Inhibitor (ThermoFisher, 4 374 966) following the standard protocol. Quantitative PCR using gene-specific primers (Table S2) was carried out using TB Green Premix Ex Taq II (Tli RNase H Plus) (Takara, RR82WR) on a LightCycler 480 system (Roche). GAPDH was used as the endogenous control, and relative gene expression was calculated using the ΔΔCt method.

### Protein purification and western blot

Cells were harvested 72 h after transfection using RIPA buffer (ThermoFisher, 89 900) following the user guide, supplemented with phosphatase (ThermoFisher, A32957) and protease inhibitors (Roche, 04693159001). Protein concentration was measured using the BCA assay (ThermoFisher, 23 227).

Equal amounts of protein (30–40 μg) were separated by SDS-PAGE (Bio-Rad, 4 561 083) and transferred to nitrocellulose membranes (Bio-Rad, 1 620 115). The membranes were blocked with Intercept tris-buffered saline (TBS) blocking buffer (LI-COR, 927–66 003) for 1 h, then incubated overnight at 4°C with primary antibodies: rabbit monoclonal [EPR8586] to PAX2 (1:500; Abcam, ab150391), rabbit polyclonal to PAX8 (1:1000; Biopat, PA030), or rabbit polyclonal to GAPDH (1:10000; Abcam, ab9483), followed by the secondary antibodies at room temperature for 1 h: IRDye 800CW Donkey anti-Rabbit IgG (1:20000; LI-COR, 926–32 213) for PAX2 and PAX8, and IRDye 680RD Donkey anti-Goat IgG (1:20000; LI-COR, 926–68 074) for GAPDH.

Blots were visualized using the LI-COR imaging system. Band intensities were quantified with ImageJ and normalized to GAPDH. Results are presented as mean ± SD, and statistical significance was determined using a *P*-value of ≤ 0.05.

### SYBR green I cell proliferation assay

600 MDCK cells or 1200 WT9–7/WT9–12 cells treated with siRNAs were plated in 96-well plates and collected after 24, 48, 72, and 96 h. After PBS wash and storage at −80°C, all plates were thawed and then 200 μl of SYBR Green I lysis buffer (50 mM EDTA, 200 mM Tris, 1% Triton X-100, and SYBR Green I (1:8000; ThermoFisher, S33102)) was added per well. Fluorescence was measured after overnight incubation at 4°C (excitation/emission: 485/535 nm), with background correction using buffer-only controls.

### Immunofluorescence staining of formalin-fixed, paraffin-embedded (FFPE) kidney tissues

For immunofluorescence staining three blocks of ADPKD tissue were collected from a single patient at Dunedin hospital, New Zealand, specifically from cystic regions of the kidney cortex. There was no mutation analysis conducted. One block of normal human kidney tissue, sourced from Christchurch Cancer Society Tissue Bank, was from normal adjacent kidney tissue of a patient with kidney cancer. All tissue collection was approved by the University of Otago Human Ethics Committee, and informed consent was obtained.

Wash with modified TBST buffer containing Ca^2+^ and Mg^2+^ was applied between each step. After blocking in animal-free blocking buffer (Vector Laboratories, SP-5035), sections were incubated with primary antibodies (rabbit monoclonal anti-PAX2 (1:30; Abcam, ab79389), rabbit polyclonal anti-PAX8 (1:100; Biopat, PA 030), and anti-LRP2/megalin (1:200; Abcam, ab76969)), Alexa Fluor 594-conjugated goat anti-rabbit IgG (H + L) secondary antibody (1:200; ThermoFisher, A11012), and FITC-conjugated lectins (*Lotus Tetragonolobus* lectin (LTL; 1:50; ThermoFisher, L32480) and soybean agglutinin (SBA; 1:200; Vector Laboratories, FL-1011-2)) sequentially. Slides were mounted using ProLong Diamond Antifade with DAPI (ThermoFisher, P36965).

### RNA library preparation and sequencing

RNA samples were sent to Auckland Genomics for library preparation and sequencing. Libraries were prepared using the Illumina Stranded mRNA Prep Kit (Illumina, 20 040 532) following the manufacturer’s instructions. Library quality and concentration were assessed using the Agilent Bioanalyzer High Sensitivity DNA Kit (Agilent, 5067–4626) and Qubit 1X dsDNA HS Assay Kit (Invitrogen, Q33230). Equimolar libraries were pooled and sequenced on a shared lane using a 300-cycle kit (2 × 150 bp paired-end reads) on the NovaSeq X Plus platform.

### Read preprocessing and alignment

Forward and reverse reads from two lanes were merged per sample. Quality control of raw and trimmed reads was conducted using FastQC (v0.11.9), and adapter trimming was performed with fastp (v0.22.0) using default parameters. Reads were aligned to the T2T-CHM13v2.0 reference genome (GCF_009914755.1) using HISAT2 (v2.2.1). SAM files were converted and sorted to BAM format using Samtools (v1.16.1). Gene-level counts were generated using FeatureCounts (Subread v2.0.7) with the following options: exon-level counting (−t exon), gene-level summarization (−g gene_id), paired-end mode (−p), and reverse-strand specificity (−s 2), using the corresponding GTF annotation file.

### Differential expression and enrichment analysis

All downstream analyses were performed in R (v4.4.1). Differential gene expression was analyzed using DESeq2 (v1.44.0) with the design formula ~ batch + condition. Significant differentially expressed genes (DEGs) were defined as those with Benjamini–Hochberg adjusted p-values (False Discovery Rate, FDR) ≤ 0.05. Overrepresentation analysis (ORA) using significant DEGs was assessed using the enrichGO function in ClusterProfiler (v4.12.6) with the biological processes (BP) terms from Gene Ontology database and org.Hs.eg.db (v3.19.1). Top5 enriched pathways were selected by adjusted P. Gene Set Enrichment Analysis (GSEA) using all genes was also performed with ClusterProfiler using the gseGO function applying the same GO BP terms. Genes were ranked by the stat value from DESeq2, with a tie-breaking adjustment using the formula: stat +1e−6 ^*^ log_2_FoldChange + 1e−9 ^*^ (−log_10_(p-value)). GO slim subset (Generic GO subset maintained by GO Consortium) was obtained from Gene Ontology (https://geneontology.org/docs/go-subset-guide/). Significant GO terms were defined as FDR ≤ 0.05. Visualizations were generated using ggplot2 (v3.5.1), GseaVis (v0.0.5), UpSetR (v1.4.0), VennDiagram (v1.7.3), and pheatmap (v1.0.12).

### External ADPKD datasets

Single-nucleus RNA sequencing (snRNA-seq) datasets (GSE185948) from human ADPKD kidneys [15] were accessed via the Kidney Interactive Transcriptomics platform (https://humphreyslab.com/SingleCell/). DEGs filtered by differential analyses were performed as described in the original publication (logFC ≥0.25, FDR ≤ 0.05). DEGs in either proximal tubule 1 (PT1), proximal tubule 2 (PT2), and connecting tubule and principal cells (CNT_PT) clusters [15] were used for investigation of *PAX2* and *PAX8* gene expression and integration with *PAX2* and *PAX8* downstream genes.

In addition, microarray data (GSE7869) comparing human ADPKD cysts and minimal cystic tissues were analysed using GEO2R. Probes without assigned gene symbols were excluded. Probes matching multiple gene symbols were also removed. In cases where the same gene symbol was linked to multiple probes showing opposite expression changes in ADPKD (i.e. some with log_2_FC > 0 and others with log_2_FC < 0), that gene was excluded from further analysis. Unique gene symbols for DEGs with FDR ≤ 0.05 were filtered to generate final gene lists for further comparison. The full analysis scripts are available at https://github.com/QzQ-702/GSE305691.

### Statistical analysis

Statistical comparisons outside of RNAseq were performed in GraphPad Prism (v10.3.1). Paired t-tests were used for two-group comparisons unless otherwise stated. One-way ANOVA followed by multiple comparisons against the control (siNC) was used for three or more groups unless otherwise stated. P ≤ 0.05 were considered statistically significant.

## Supplementary Material

Original_article_format_revised_supl_tables_and_figures_ddaf205

## Data Availability

The RNA-seq datasets of PAX2 and PAX8 knockdowns generated during this study are available in the NCBI Gene Expression Omnibus (GEO) repository (https://www.ncbi.nlm.nih.gov/geo/) under accession number GSE305691. Two external datasets analysed in this study are publicly available: snRNA-seq data from human ADPKD kidneys (GSE185948, as described in the original publication [15]; data visualization accessible via the Kidney Interactive Transcriptomics platform: https://humphreyslab.com/SingleCell/) and microarray data comparing human ADPKD cysts with minimal cystic tissues (GSE7869) [45]. All datasets are publicly accessible, and further details are provided in the corresponding references.
