# Supplementary material for: PAX gene expression in autosomal dominant polycystic kidney disease contributes to cyst expansion and regulates a gene network associated with cyst growth
Source: Hum Mol Genet. 2026 Jan 8;35(3):ddaf205. doi: 10.1093/hmg/ddaf205 (PMC13158232; doi:10.1093/hmg/ddaf205)
Supplement: Original_article_format_revised_supl_tables_and_figures_ddaf205 [file original_article_format_revised_supl_tables_and_figures_ddaf205.pdf]

Supplementary Tables:

Table S1 siRNA sequences used in this study.

siRNA target	Name	Sequence (5'->3')	Manufacturer
Non-targeting control	siNC	UUCUCCGAAGGUGUCACGUUU	Shanghai Genepharma Co., Ltd.
PAX2	si*PAX2*	GAAGUCAAGUCGAGUCUAUUU	
PAX8	si*PAX8*	CAUCCGGCCUGGAGUGAUA	

Table S2 The primer sequences used in this study

Name	Sequence (5'->3')	Species specificity
human_*GAPDH*_F	CTCAAGATCATCAGCAATGCC	Human
human_*GAPDH*_R	GGTCATGAGTCCTTCCACGATAC	Human
dog_*GAPDH*_F	AACATCATCCCTGCTTCCAC	Dog
dog _*GAPDH*_R	GGCAGGTCAGATCCACAACT	Dog
*PAX2*_F	CCTGGCCACACCATTGTTC	Dog, Human
*PAX2*_R	TCACGTTTCCTCTTCTCACCAT	Dog, Human
human_*PAX8*_F	TGAGGGCGTCTGTGACAATG	Human
human_*PAX8*_R	CGGGACTCAGGGACTTGGT	Human
dog_*PAX8*_F	TCAGCAAGATCCTTGGCAGG	Dog
dog_*PAX8*_R	CATGGTAGGGTTCTGCCGTT	Dog

Supplementary Figures:

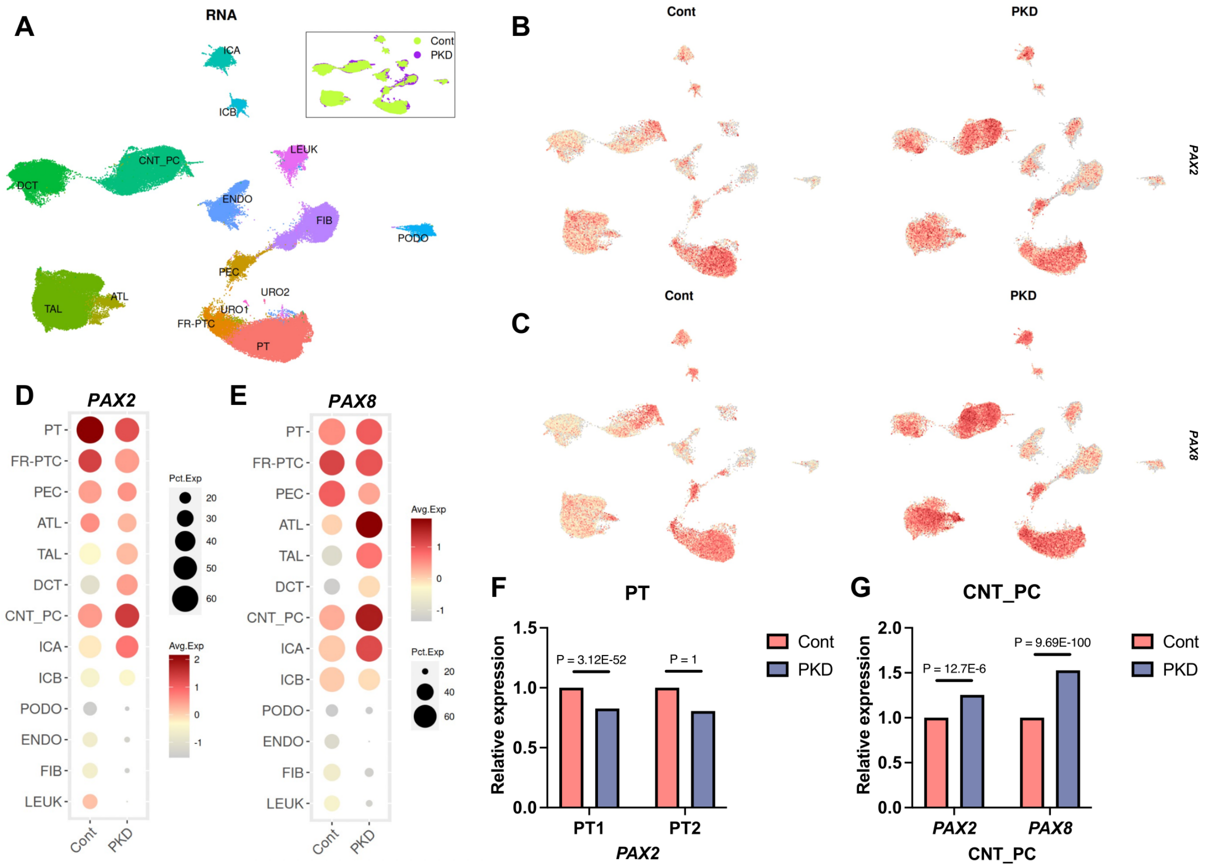

Figure S1. ***PAX2*** **and** ***PAX8*** **expression in human ADPKD**

(A) UMAP plot of integrated snRNA-seq datasets with annotation by cell type. The small UMAP plots on the top right was annotated by disease condition. PT proximal tubule, PEC parietal epithelial cells, TAL thick ascending limb of Henle's loop, DCT distal convoluted tubule, CNT_PC connecting tubule and principal cells, ICA Type A intercalated cells, ICB Type B intercalated cells, PODO podocytes, ENDO endothelial cells, FIB fibroblasts, LEUK leukocytes, URO uroepithelium. **(B-C)** UMAP displaying enrichment of *PAX2* (B) or *PAX8* (C) in snRNA-seq dataset for ADPKD or control kidneys. **(D-E)** Dot plots of snRNA-seq dataset showing gene expression patterns of *PAX2* (**D**) or *PAX8* (**E**) for ADPKD or control kidneys. The diameter of the dot corresponds to the proportion of cells expressing the indicated gene and the intensity of the dot corresponds to average expression relative to all cell types. **(F)** Expression level of *PAX2* between ADPKD and control kidneys in PT1 and PT2 cell types from snRNA-seq dataset. (**G**) Expression level of *PAX2* and *PAX8* between ADPKD and control kidneys in the CNT_PC cell type from snRNA-seq dataset.

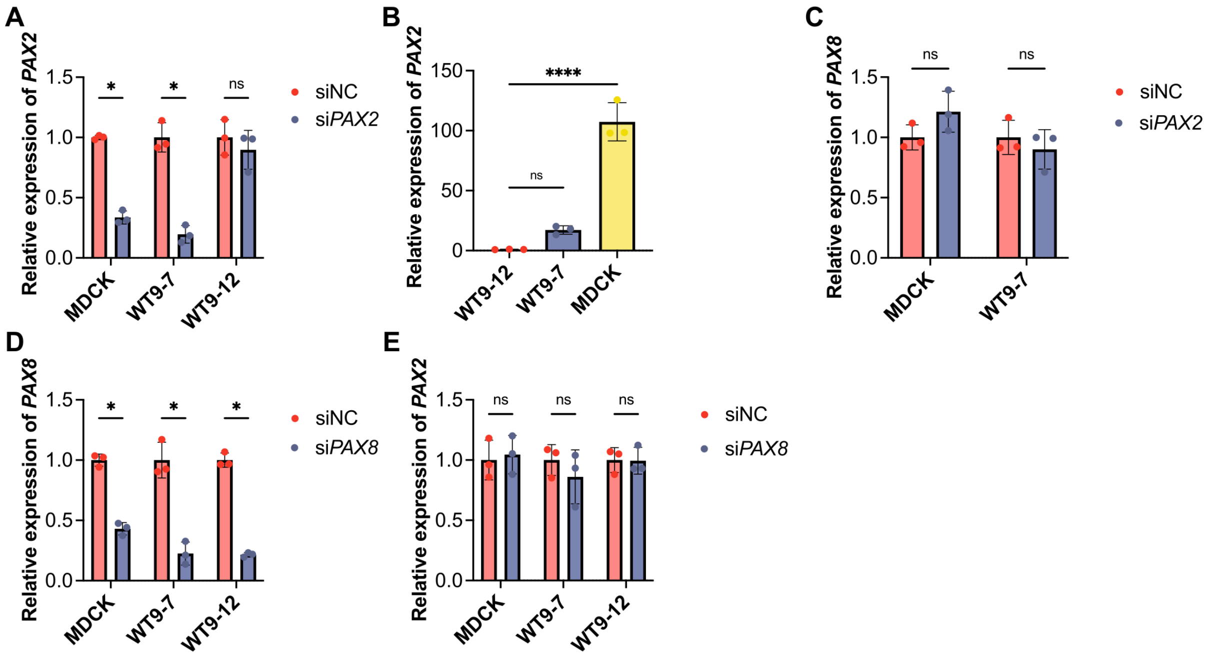

Figure S2. ***PAX2*** **and** ***PAX8*** **expression measured by RT-qPCR**

(A) PAX2 mRNA expression after transfection of 10 nM si*PAX2* or siNC in MDCK, WT9-7, and WT9-12 cells. **(B)** Endogenous *PAX2* mRNA expression in WT9-12, WT9-7, and MDCK cells, normalized to expression in WT9-12 cells. **(C)** *PAX8* mRNA expression after transfection of 10 nM si*PAX2* or siNC in MDCK, WT9-7, and WT9-12 cells. Expression levels were normalized to siNC. **(D)** *PAX2* mRNA expression 24 hours after transfection of 10 nM si*PAX8* or siNC in MDCK, WT9-7, and WT9-12 cells, normalized to siNC. **(E)** *PAX2* mRNA expression 24 hours after transfection of 10 nM si*PAX8* or siNC in MDCK, WT9-7, and WT9-12 cells, normalized to siNC. Mean ± SD from n = 3 independent experiments. One-way ANOVA. ns: not significant; *P ≤ 0.05; ****P ≤ 0.0001.

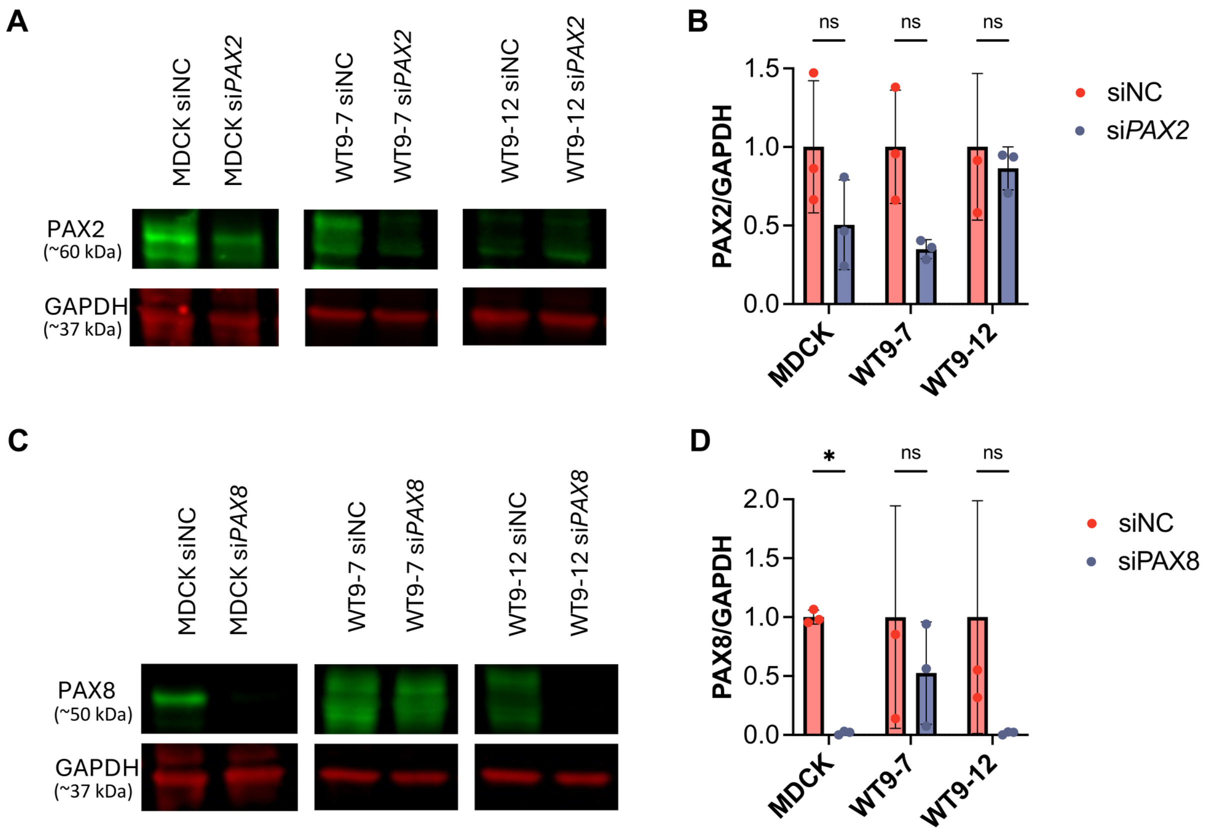

Figure S3. PAX2 and PAX8 protein expression by Western blot

(A) PAX2 protein expression 72 hours after transfection of 10 nM siPAX2 or siNC in MDCK, WT9-7, and WT9-12 cells. **(B)** PAX8 protein expression 72 hours after transfection of 10 nM siPAX8 or siNC in MDCK, WT9-7, and WT9-12 cells. **(C-D)** Quantification of PAX2 (A) and PAX8 (B) protein levels by analyzing western blot bands. Band intensities were quantified using ImageJ and normalized to the average of GAPDH in siNC. Mean ± SD from n = 3 biological replicates. Multiple paired t-tests. ns: not significant; *P ≤ 0.05.

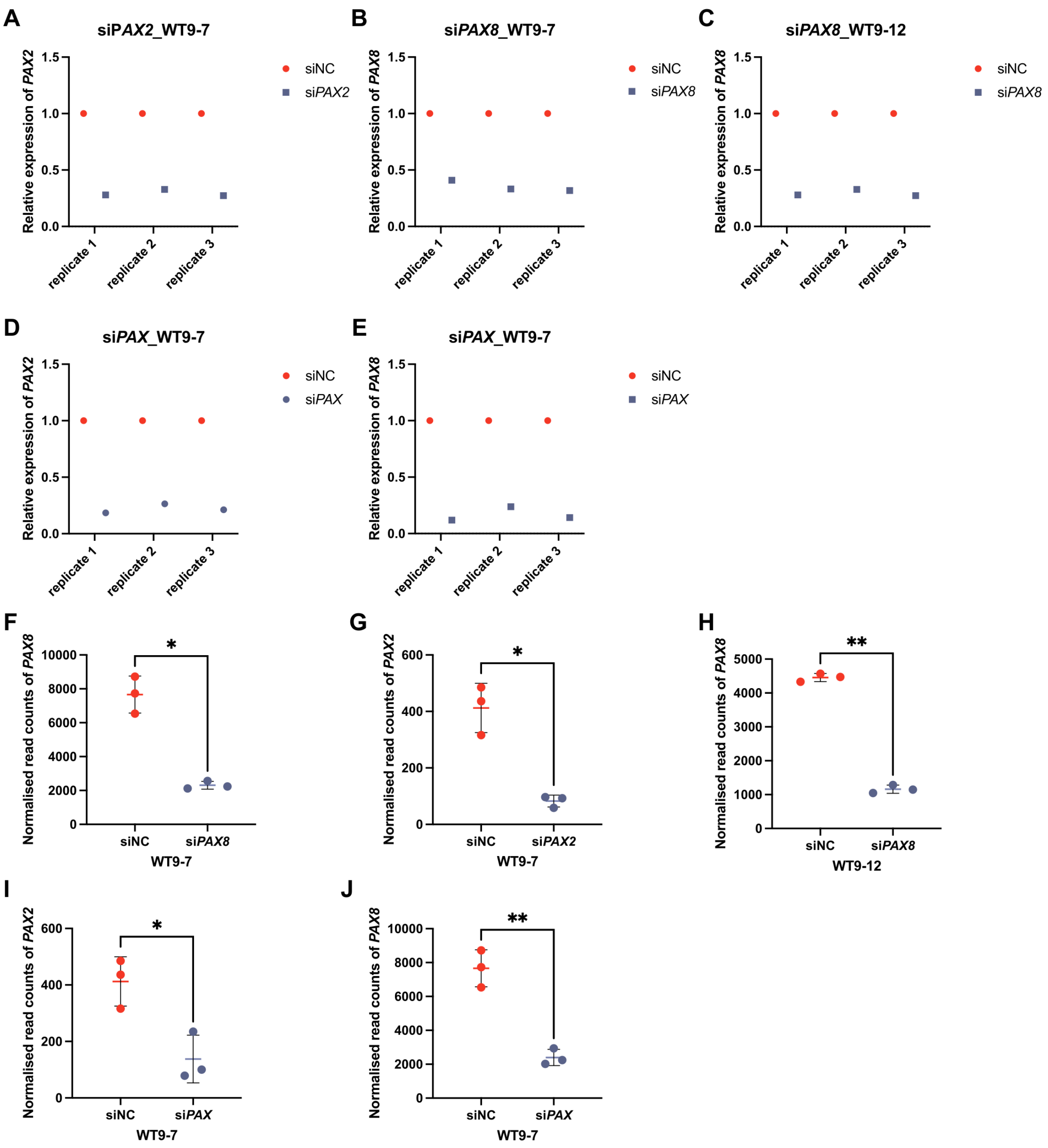

Figure S4. *PAX2* and *PAX8* knockdown efficiency validated by RT-qPCR and RNA-seq. **(A–E)** mRNA expression levels of *PAX2* and *PAX8* assessed by RT-qPCR 24 hours post-transfection in three independent knockdown experiments. (A) *PAX2* expression in WT9-7 cells after si*PAX2* or siNC; (B) *PAX8* expression in WT9-7 after si*PAX8* or siNC; (C) *PAX8* expression in WT9-12 after si*PAX8* or siNC; (D–E) *PAX2* (D) and *PAX8* (E) in WT9-7 cells after combined si*PAX2*+si*PAX8* (si*PAX*) or siNC. **(F–J)** RNA-seq-based quantification of *PAX2* and *PAX8* expression in the same experimental groups as above. Read counts were normalized using DESeq2 after batch correction. si*PAX* refers to dual knockdown with si*PAX2* and si*PAX8*. Paired t-tests, mean ± SD. *P ≤ 0.05; **P ≤ 0.01.

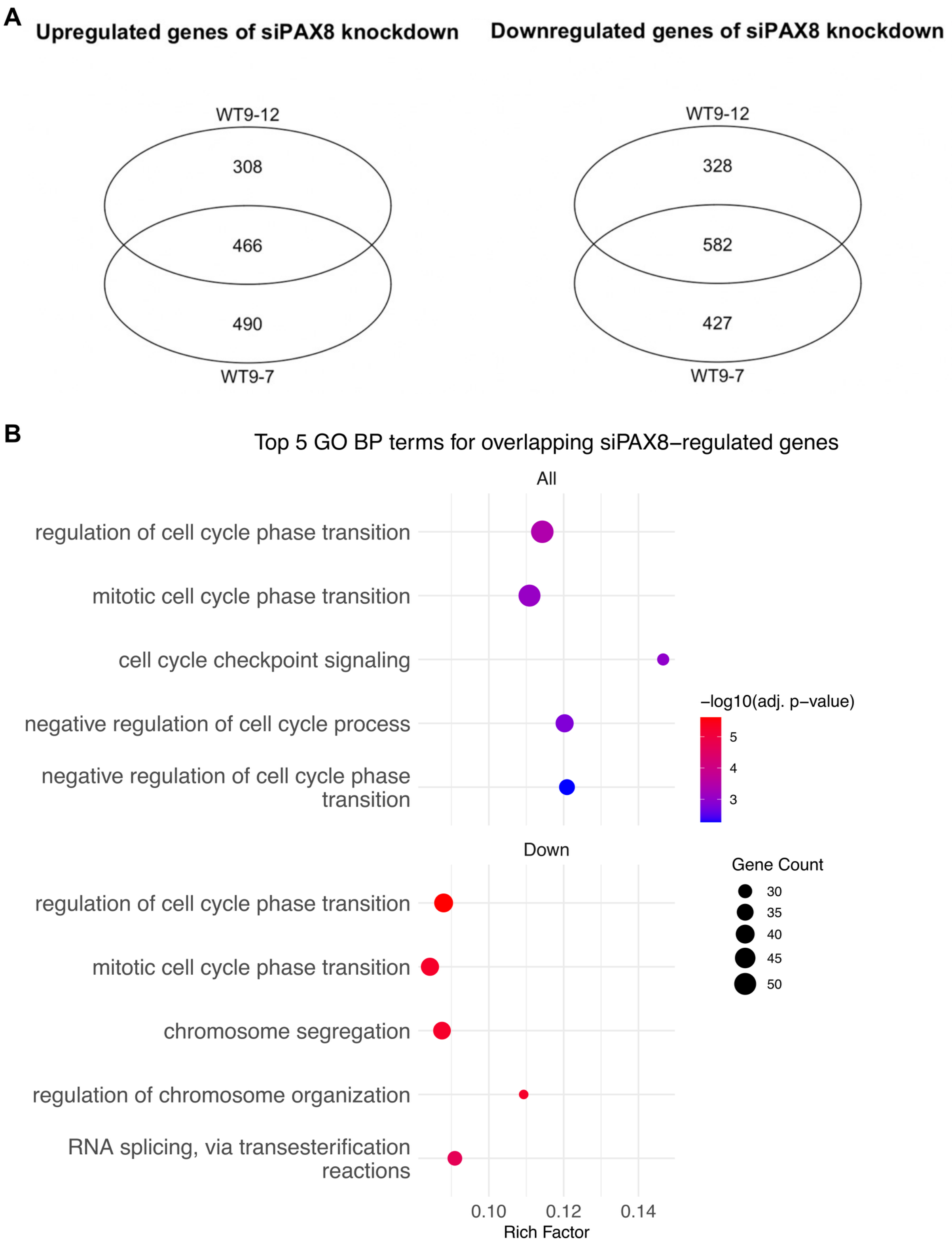

Figure S5. Gene ontology enrichment analysis of *PAX2* and *PAX8* knockdown ADPKD cells
(A) Venn plot of overlapping genes of *PAX8* knockdown in human ADPKD WT9-7 and WT9-12 cells. **(B)** Over-representation analysis (ORA) of overlapping differentially expressed genes after siPAX8 knockdown in WT9-7 and WT9-12 cells. Enriched Gene Ontology (GO) Biological

Process (BP) terms are shown for all dysregulated genes (top) and downregulated (middle) genes. No enriched Go terms for upregulated (Up). Pathways are ranked by adjusted P value (FDR ≤ 0.05). “Gene Count” indicates the number of DEGs associated with each GO term.

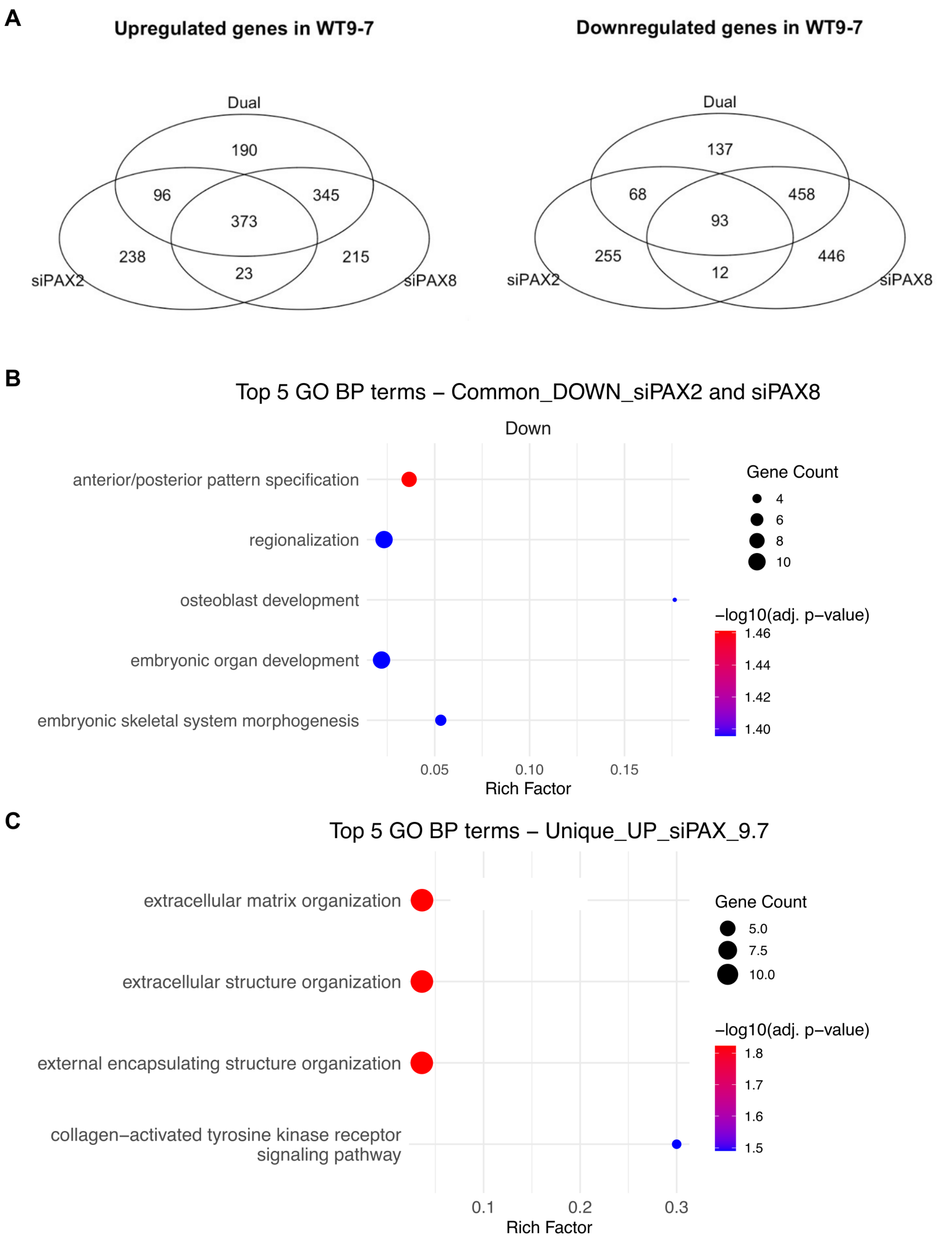

Figure S6. Extracellular matrix–related processes are enriched in uniquely upregulated genes following dual *PAX2* and *PAX8* knockdown in WT9-7 cells

(A) Venn diagrams showing the number of significantly upregulated (left) and downregulated (right) differentially expressed genes (DEGs) in WT9-7 cells following si*PAX2*,

si*PAX8*, or dual knockdown (si*PAX*), relative to siNC control. The dual knockdown resulted in 190 uniquely upregulated and 137 uniquely downregulated genes. **(B)** Over-representation analysis (ORA) of the 190 uniquely upregulated DEGs in si*PAX*. Enriched Gene Ontology (GO) Biological Process (BP) terms, particularly those related to extracellular matrix organization and remodeling, are shown. No enriched terms were identified for the uniquely downregulated genes or for the combined set of all uniquely dysregulated genes (upregulated and downregulated). Pathways are ranked by adjusted P value (FDR ≤ 0.05). "Gene Count" indicates the number of DEGs associated with each GO term.
